# Pricing of tobacco products during, and after, the introduction of standardized packaging: an observational study of retail price data from independent and convenience (small) retailers in the United Kingdom

**DOI:** 10.1111/add.14488

**Published:** 2018-12-16

**Authors:** Nathan Critchlow, Martine Stead, Crawford Moodie, Kathryn Angus, Douglas Eadie, Anne Marie MacKintosh

**Affiliations:** ^1^ Institute for Social Marketing, and UK Centre for Tobacco and Alcohol Studies, Faculty of Health Sciences and Sport, University of Stirling Scotland, UK; ^2^ Centre for Tobacco Control Research, Faculty of Health Sciences and Sport University of Stirling Scotland, UK

**Keywords:** Plain packaging, price, standardized packaging, tobacco, tobacco marketing, tobacco price strategy

## Abstract

**Background and aims:**

Tobacco companies claimed that standardized packaging, phased in/introduced May 2016–May 2017, would reduce prices and increase consumption. We: (1) describe changes in price‐per‐cigarette and price‐per‐gram during, and after, the introduction of standardized packaging; (2) describe price changes by cigarette price segment; and (3) analyse price changes by stage of implementation.

**Design:**

An observational study, using electronic point‐of‐sale data, monitored price trends in three periods: (1) May–September 2016, start of transition period; (2) October 2016–May 2017, when fully branded and standardized products were sold and duty escalators implemented; and (3) June–October 2017, when standardized packaging was mandatory.

**Setting:**

United Kingdom.

**Participants:**

Small retailers (*n* = 500) stratified by region and deprivation. Data were monitored for 20 leading fully branded tobacco products [15 factory‐made cigarettes (FMC), 5 roll‐your‐own (RYO)] and their standardized equivalents.

**Measurement:**

Price‐per‐cigarette and price‐per‐gram, based on monthly average Recommended Retail Price (RRP) and actual sale price, adjusted for inflation using the Consumer Pricing Index (CPIH). Net changes (£GBP, %) were analysed by product type (FMC versus RYO) and FMC price segment (value, mid‐price, premium).

**Findings:**

Between May 2016 and October 2017, the average inflation‐adjusted RRP/price‐per‐cigarette and price‐per‐gram increased for FMC (all price segments) and RYO. For example, sales price‐per‐cigarette increased +4.64%, with the largest increases for value (+6.81%), premium (+5.32%) and mid‐price FMCs (+3.30%). Net sales price‐per‐cigarette and price‐per‐gram increases were largest in period 2, when fully branded and standardized products were sold and duty escalators were implemented (FMC = +4.70%; RYO = +3.75%). There were small decreases in sales price‐per‐cigarette and price‐per‐gram once standardized packaging became mandatory (FMC = –1.14%; RYO = –0.88%).

**Conclusion:**

In the United Kingdom, the price of leading roll‐your‐own and factory‐made cigarette brands sold by small retailers increased as standardized packaging was phased in, with increases larger than expected through duty escalation.

## Introduction

Price is a key component of tobacco marketing strategy 1, 2, 3 and an important driver of smoking behaviour 4, 5, 6, 7. Tobacco companies contend that the introduction of standardized packaging in the United Kingdom would leave price as the only marketing lever, leading to greater competition on price and, consequently, increased affordability and consumption 8, 9, 10, 11. The Standardized Packaging of Tobacco Products Regulations 2015 and Tobacco and Related Products Regulations 2016 (which transposed into UK law the Tobacco Products Directive), came into force on 20 May 2016, with a 12‐month transition period. The legislation, referred to hereafter as standardized packaging, requires factory‐made cigarettes (FMC) and roll‐your‐own (RYO) tobacco to be sold in drab brown packs with pictorial warnings covering at least 65% of primary surfaces and text warnings covering at least 50% of secondary surfaces 12. It also prohibits price‐marking on packs and sets a minimum pack size of 20 cigarettes for FMC and 30 g for RYO.

Tobacco companies argue that standardized packaging will make price the sole identifiable product feature 13, leading companies to lower prices to remain competitive and retain market share, and that increased affordability would increase consumption, including among price‐sensitive young consumers 8, 9, 10, 11. It has also been suggested that removing features which distinguish product quality and origin may result in ‘down‐trading’ to cheaper products, which offer a more affordable price‐per‐stick 14, 15, and consistent with this consumer surveys in Australia have found an increase in the use of value cigarette brands and RYO post‐standardized packaging 16, 17. Whether this is a direct consequence of the legislation is not clear, as research in Australia has also found that tobacco companies revised their brand strategies in anticipation of greater price sensitivity among consumers, including offsetting price rises (i.e. absorbing price increases generated by taxation), offering larger pack sizes with a more affordable price‐per‐cigarette, and more value and super‐value brand variants 18, 19, 20, 21. Similar trends have been observed in the United Kingdom, and tobacco companies have emphasized the importance of recommended retail price (RRP) to retailers 22, 23, 24.

Tobacco companies are known to alter their prices in response to changes in policy and taxation, including absorbing tax increases on the cheapest cigarettes to preserve price competitiveness (under‐shifting) and charging extra for more expensive cigarettes to maximize profits (over‐shifting) 14, 25, 26. Research from Australia, however, does not support tobacco companies’ claim that standardized packaging would increase the affordability of tobacco. A study of RRPs from leading tobacco companies published in trade magazines found that inflation‐adjusted RRPs‐per‐cigarette and per‐gram increased in the year after the legislation was passed (November 2011–November 2012) and the year after implementation (November 2012–November 2013) 27. Increases occurred for both FMC and RYO, and among value, mainstream (mid‐price) and premium brands. Inflation‐adjusted increases were also evident in the advertised prices in retailers for the most prominently promoted products (i.e. those listed at the top of advertised price lists) and the lowest‐priced products 28. There was no evidence that the increased availability and use of value brands following the introduction of standardized packaging was associated with increased consumption 16.

Tobacco taxation provides important context to price changes; for example, to understand whether tobacco companies absorb increases in tax payable on their products, pass these onto consumers or introduce additional increases above tax changes 25. In the United Kingdom, FMC taxation comprises two components: a duty per 1000 cigarettes (with an annual escalator of 2% above inflation) and an *ad valorem* duty (16.5% of the retail price) 29, 30. Taxation for RYO represents a single duty per‐kilogram, with an annual escalator of 2% above inflation 30. Both FMC and RYO products are subject to value‐added tax (VAT) (20% of sales price). Two changes to taxation occurred during the introduction of standardized packaging. First, duties for FMC and RYO were raised through their annual escalators in March 2017, 2 months before standardized packaging became mandatory 31. Secondly, a minimum excise tax (MET) was introduced to create a ‘floor price’ for FMCs (i.e. selling below would mean that duty payable would exceed sales revenue) to tackle the availability of ultra‐low‐price cigarettes 32. Under the MET, the tax payable for FMCs became the higher of typical duty (per 1000 cigarettes plus *ad valorem* duty) or the minimum duty threshold (initially £268.63 per 1000 cigarettes) 32. The MET was introduced when standardized packaging became mandatory (20 May 2017).

This study used monthly retail price data from small retailers to: (1) describe changes in the average price‐per‐cigarette and price‐per‐gram during, and after, the introduction of standardized packaging; (2) describe price changes by cigarette price segment (value, mid‐price and premium); and (3) examine variation in price change by stage of implementation (start of the transition period, during the transition period when both fully branded and standardized packs were sold and annual duty escalators were implemented, and when standardized packaging became mandatory). We explore trends in small retailers, an important group to investigate as more than half consider tobacco important to profit and footfall 33 and because they account for more than half the volume of cigarette sales in the United Kingdom 34.

## Methods

### Design and observation periods

An observational study using monthly Electronic Point of Sale (EPoS) data (i.e. the hardware and software used to process sales and manage stock) monitored price trends in small retailers in England, Scotland and Wales, as part of a project exploring trends in product availability and pricing during the introduction of standardized packaging 35, 36, 37. Data were collected for 18 months (May 2016–October 2017), and divided into three periods to account for trends in product availability, stage of implementation and tax changes (Table 1).

**Table 1:** Observation periods and characteristics of product availability, tobacco duty and implementation of legislation.

Study period	Months in period	Trends in product availability, tobacco duty and legislation implementation
Period 1 (P1)	May 2016–September 2016	• First 5 months of transition to standardized packaging • 2 months after annual tax duty escalators were implemented in March 2016 • Only fully branded tobacco (non‐compliant) products sold in small retailers 36
Period 2 (P2)	October 2016–May 2017	• Final 7 months of transition to standardized packaging • Annual tax duty escalators were implemented in March 2017 • Both fully branded (non‐compliant) and standardized (compliant) tobacco products sold in small retailers 36
Period 3 (P3)	June 2017–October 2017	• Standardized packaging mandatory • Minimum pack sizes mandatory (20 FMC; 30 g RYO) • The Minimum Excise Tax is introduced for FMCs

FMC = factory‐made cigarettes; RYO = roll‐your‐own.

### Retailer sample

Data were obtained from The Retail Data Partnership Ltd (TRDP), who supply EPoS systems to approximately 2300 small retailers in the United Kingdom. The TRDP database includes both symbol group‐affiliated stores and independent stores 38. A stratified random sample of stores (*n =* 500) was selected for monitoring (300 in England, 100 in Scotland, 100 in Wales), with stores stratified by region and indices of deprivation. Further details on stratification and replacement for attrition are reported elsewhere 36.

### Tobacco products monitored

All tobacco products were monitored through Universal Product Codes (i.e. barcodes). Forty tobacco products were selected, comprising 20 of the best‐selling fully branded products (15 FMC 20‐cigarette packs and five RYO 25‐g packs, or nearest size equivalent) and the 20 standardized products which replaced them (Table 2). Data on cumulative sales value (£) from March 2015 to March 2016 were used to select best‐selling products at baseline. FMCs were classified into value, mid‐price and premium price segments using the average price‐per‐stick from March 2015 to March 2016, with segmentation based on thresholds reported in the retail and industry trade press 39. Only a small number of RYO products were monitored, and no price segmentation was used. If a fully branded product was sold in both a price‐marked (i.e. RRP printed on the cellophane) and non‐price‐marked pack, we received information on each variant separately and combined average. Price‐marking was not permitted for standardized products. One RYO fully branded product (John Player Special Silver 25 g) was discontinued during the phase‐in period, and thus only 19 standardized products were monitored (four RYO). Details on sales trends are reported elsewhere 36, 37.

**Table 2:** The fully branded products monitored from May 2016 and the replacement compliant products, by price segment.

Fully branded and non‐compliant^a^	Standardized and compliant^b^
Value cigarettes^c^	
Carlton King Size 19 sticks	Carlton King Size Red 20 sticks
Carlton Superkings 19 sticks	Carlton Superkings Red 20 sticks
Players King Size 18 sticks	JPS Players King Size Real Red 20 sticks
Players Superkings 18 sticks	JPS Players Superkings Real Red 20 sticks
Rothmans Superkings Value Blue 18 sticks	Rothmans Superkings Value Blue 20 sticks
Mid‐price cigarettes^c^	
John Player Special King Size Blue 19 sticks	JPS King Size Real Blue 20 sticks
Lambert & Butler King Size 20 sticks	Lambert & Butler King Size Original Silver 20 sticks
Lambert & Butler King Size Blue 19 sticks	L&B Blue King Size Real Blue 20
Mayfair King Size 19 sticks	Mayfair King Size 20 sticks
Richmond King Size 19 sticks	Richmond King Size Real Blue 20 sticks
Richmond Superkings 19 sticks	Richmond Superkings Real Blue 20 sticks
Rothmans King Size Value Blue 18 sticks	Rothmans King Size Value Blue 20 sticks
Sterling King Size Dual 17 sticks	Sterling King Size Dual 20 sticks
Premium cigarettes^c^	
Benson & Hedges Gold 20 sticks	Benson & Hedges King Size Gold 20 sticks
Marlboro King Size Gold 20 sticks	Marlboro King Size Gold 20 sticks
Roll‐your‐own tobacco	
Amber Leaf Rolling Tobacco 25 g	Amber Leaf Original Rolling Tobacco 30 g
Gold Leaf 25 g (RYO)	Gold Leaf JPS Quality Blend 30 g
Golden Virginia Classic 25 g	Golden Virginia The Original 30 g
Golden Virginia Smooth 25 g	Golden Virginia Bright Yellow 30 g
John Player Special Silver 25 g	No standardized and compliant equivalent

a Non‐compliant = fully branded packaging, no minimum pack size and price‐marking permitted on product packaging.

b Compliant = standardized packaging, minimum pack sizes, no price‐marking permitted on product packaging and no misleading names.

c FMC price segment: value = ≤ £0.35 per‐cigarette; mid‐price = £0.36–0.43 per‐cigarette; premium = ≥ £0.44 per‐cigarette, based on March 2015–March 2016 sales data 39. FMC = factory‐made cigarettes; RYO = roll‐your‐own.

### Price measures

RRP represented the default sales value (£GBP) set on the EPoS system. RRPs were periodically downloaded from wholesaler price databases to each retailer's EPoS system, although these prices could be manually adjusted by retailers to increase profitability or implement local‐level price offers. Sales price represented the actual value recorded at the point of transaction (£GBP). For each product, we received a monthly average RRP and sales price for each retailer (inclusive of VAT at 20%).

### Analysis

Data were analysed using SPSS version 23 and Microsoft Excel. In each month, the nominal average RRP and sales price (i.e. unadjusted for inflation and tax) were calculated for each product across the retailer sample, with 5% trimmed means used to exclude outlying values from manual EPoS errors. The nominal average RRP and sales price for each product were then adjusted to October 2017 prices using the ‘all items’ Consumer Prices Index, including owner‐occupiers’ housing costs (CPIH), the lead measure of inflation in the United Kingdom 40, 41. Inflation increased 3.6% over the 18 months of observation (CPIH Index range = 100.8–104.4; base year = 2015). Inflation‐adjusted prices were calculated by multiplying the nominal average RRP and sales price for each product by the CPIH ‘all items’ Index for October 2017 divided by the Index value for each reference month.

The monthly RRP‐per‐cigarette/gram and sales price‐per‐cigarette/gram was calculated for each product by dividing the average inflation‐adjusted RRP and sales price by pack size. The average RRP/price‐per‐cigarette was calculated across all FMC products and in each price segment (value, mid‐price, premium). The average RRP/price‐per‐gram was calculated across the RYO category, without segmentation. In each month, a product was only included in the average RRP/price‐per‐cigarette or per‐gram if it had been sold by at least 1% of the retailer sample (*n* = 5). If a product was available in a price‐marked and non‐price‐marked variant, prices‐per‐cigarette and prices‐per‐gram were computed using the combined average.

Trends in RRP‐per‐cigarette/gram and sales price‐per‐cigarette/gram were analysed through net price changes (£GBP and %) within each period, throughout the transition year (P1 and P2 combined), and throughout the observation period (P1–P3, combined). To enhance sensitivity, net changes were calculated with cigarette and gram prices to four decimal places. Changes in estimated pack cost were computed by multiplying the RRP/price‐per‐cigarette and RRP/price‐per‐gram by the new minimum pack sizes (20 FMC or 30 g RYO) and subtracting the difference between the end and start of each period. We also monitored the price difference between FMC categories by comparing the percentage difference between RRP/price‐per‐cigarette for value versus mid‐price, and for mid‐price versus premium, at the beginning (May 2016) and end of the study (October 2017).

## Results

### Trends in RRP‐per‐gram and RRP‐per‐cigarette

**Figure 1:**
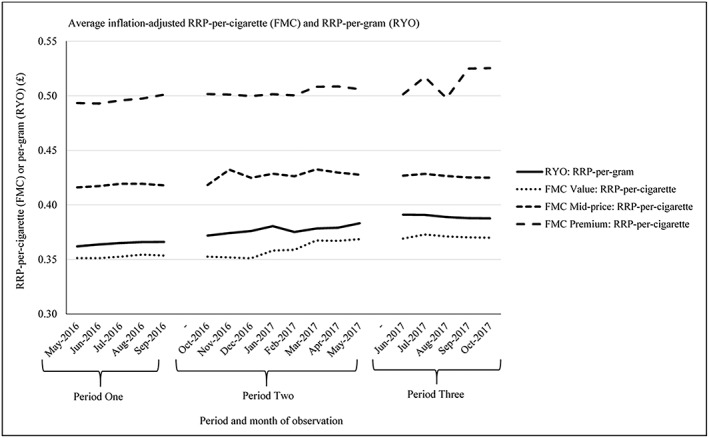
RRP‐per‐cigarette and per‐gram, based on inflation‐adjusted average RRP, by product type and FMC price segment. RRP = recommended retail price; FMC = factory‐made cigarettes; RYO = roll‐your‐own

Between May 2016 and October 2017, the average inflation‐adjusted RRP‐per‐gram for RYO increased +7.07%, equivalent to a £0.77 increase for a 30‐g RYO pack (Table 3) (Fig. 1). The largest net increase in average RRP‐per‐gram occurred in P2 (+3.07%). There was a small net decrease in RRP‐per‐gram in P3 (−0.89%).

**Table 3:** Inflation‐adjusted RRP‐per‐cigarette (FMC) and RRP‐per‐gram (RYO) across the three study periods.

Period	FMC				RYO
	Overall	Value	Mid‐price	Premium	Overall
Period 1 (May 2016–September 2016)					
RRP‐per‐cigarette/gram in first month (£)	0.40	0.35	0.42	0.49	0.36
Net change (£)	+0.0027	+0.0023	+0.0018	+0.0076	+0.0040
Net change (%)	+0.67	+0.65%	+0.43%	+1.54	+1.10
Period 2 (October 2016 – May 2017)					
RRP‐per‐cigarette/gram in first month (£)	0.41	0.35	0.42	0.50	0.37
Net change (£)	+0.0143	+0.0160	+0.0094	+0.0045	+0.0114
Net change (%)	+3.51	+4.54	+2.25	+0.90%	+3.07
Period 3 (June 2017–October 2017)					
RRP‐per‐cigarette/gram in first month (£)	0.42	0.37	0.43	0.50	0.39
Net change (£)	−0.0020	+0.0008	−0.0019	+0.0240	−0.0035
Net change (%)	−0.47	+0.22	−0.45%	+4.79%	−0.89
Transition year (May 2016–May 2017)					
RRP‐per‐cigarette/gram in first month (£)	0.40	0.35	0.42	0.49	0.36
Net change (£)	+0.0170	+0.0174	+0.0116	+0.0127	+0.0212
Net change (%)	+4.20	+4.95	+2.79%	+2.57	+5.86
18 months (May 2016–October 2017)					
RRP‐per‐cigarette/gram in first month (£)	0.40	0.35	0.42	0.49	0.36
Net change (£)	+0.0152	+0.0187	+0.0088	+0.0320	+0.0256
Net change (%)	+3.75	+5.32	+2.11	+6.49%	+7.07

In each net change calculation, the denominator was average inflation‐adjusted RRP‐per‐stick/gram in the first month of each period.

All prices adjusted to October 2017 prices using the Consumer Pricing Index (CPIH). RRP = recommended retail price; FMC = factory‐made cigarettes; RYO = roll‐your‐own.

Between May 2016 and October 2017, the average inflation‐adjusted RRP‐per‐cigarette for FMCs increased +3.75%, equivalent to a £0.30 increase for a 20 FMC pack (Table 3) (Fig. 1). The net increase was highest for premium FMCs (+6.49%, £0.64 increase per 20 FMC), then value (+5.32%, £0.37 increase per 20 FMC) and mid‐price FMCs (+2.11%, £0.18 increase per 20 FMC). The net increase in average RRP‐per‐cigarette was largest in P2 (+3.51%), with the increases highest for value (+4.45%) and mid‐price FMCs (+2.25%). There was a net decrease in RRP‐per‐cigarette in P3 (−0.47%) driven by declines for mid‐priced FMCs (−0.45%), given that there were net increases for value (+0.22%) and premium FMCs (+4.79%).

In October 2017 (end of P3), when only standardized products were sold, the average RRP‐per‐cigarette for mid‐price FMCs was +14.87% higher than for value FMCs, which is lower than the corresponding difference in May 2016 (start of P1), when only fully branded products could be sold (+18.48%). Conversely, the average RRP‐per‐cigarette for premium FMCs was +23.65% higher than mid‐price FMCs, which was greater than the corresponding difference in May 2016 (start of P1) (+18.58%).

### Trends in sales price‐per‐gram and sales price‐per‐cigarette

**Figure 2:**
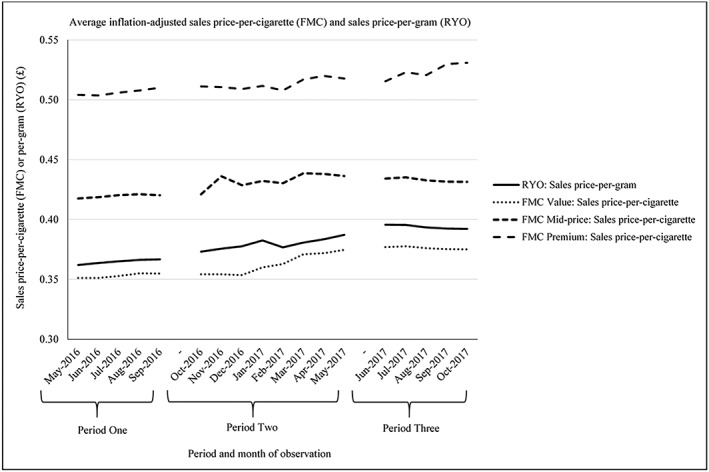
Sales‐per‐cigarette and per‐gram, based on inflation‐adjusted average sales price, by product type and factory‐made cigarettes (FMC) price segment. FMC=factory‐made cigarettes; RYO=roll‐your‐own

Between May 2016 and October 2017, the average inflation‐adjusted sales price‐per‐gram for RYO products increased +8.34%, equivalent to £0.91 increase for a 30 g pack (Table 4) (Fig. 2). The largest net increase in average price‐per‐gram occurred in P2 (+3.75%). There was a net decrease in price‐per‐gram in P3 (−0.88%).

**Table 4:** Inflation‐adjusted sales price‐per‐cigarette (FMC) and sales price‐per‐gram (RYO) throughout the three study periods.

Period	FMC				RYO
	Overall	Value	Mid‐price	Premium	Overall
Period 1 (May 2016–September 2016)					
Sales price‐per cigarette/gram in first month (£)	0.41	0.35	0.42	0.50	0.36
Net change (£)	+0.0035	+0.0037	+0.0027	+0.0060	+0.0047
Net change (%)	+0.86	+1.05	+0.65	+1.19	+1.30
Period 2 (October 2016–May 2017)					
Sales price‐per cigarette/gram in first month (£)	0.41	0.35	0.42	0.51	0.37
Net change (£)	+0.0193	+0.0204	+0.0152	+0.0068	+0.0140
Net change (%)	+4.70	+5.76	+3.61	+1.33	+3.75
Period 3 (June 2017–October 2017)					
Sales price‐per cigarette/gram in first month (£)	0.43	0.38	0.43	0.52	0.40
Net change (£)	−0.0049	−0.0019	−0.0028	+0.0154	−0.0035
Net change (%)	−1.14	−0.50	−0.64	+2.99	−0.88
Transition year (May 2016–May 2017)					
Sales price‐per cigarette/gram in first month (£)	0.41	0.35	0.42	0.50	0.36
Net change (£)	+0.0231	+0.0235	+0.0187	+0.0139	+0.0252
Net change (%)	+5.68	+6.69	+4.48	+2.76	+6.96
18 months (May 2016–October 2017)					
Sales price‐per cigarette/gram in first month (£)	0.41	0.35	0.42	0.50	0.36
Net change (£)	+0.0189	+0.0239	+0.0138	+0.0268	+0.0302
Net change (%)	+4.64	+6.81	+3.30	+5.32	+8.34

In each net change calculation, the denominator was average inflation‐adjusted sales price‐per‐stick/gram in the first month of each period

All prices adjusted to October 2017 prices using the Consumer Pricing Index (CPIH). FMC = factory‐made cigarettes; RYO = roll‐your‐own.

Between May 2016 and October 2017, the average inflation‐adjusted sales price‐per‐cigarette for FMCs increased +4.64%, equivalent to a £0.38 increase on a 20 FMC pack (Table 4) (Fig. 2). In monetary terms, the net increase was highest for premium FMCs (+5.32%, £0.54 increase per 20 FMCs), followed by value (+6.81%, £0.48 increase per 20 FMCs) and mid‐price FMCs (+3.30%, £0.28 increase per 20 FMCs), although value FMCs had the largest relative (percentage) increase. The largest net increase in price‐per‐cigarette occurred in P2 (+4.70%), with increases highest for value (+5.76%) and mid‐price FMCs (+3.61%). There was an overall net decrease in the average price‐per‐cigarette in P3 (−1.14%), with net decreases for mid‐priced (−0.64%) and value FMCs (−0.50%), but a net increase for premium FMCs (+2.99%).

In October 2017 (end of P3), the average sales price‐per‐cigarette for mid‐price FMCs was +15.00% higher than for value FMCs, which is lower than the corresponding difference in May 2016 (start of P1) (+18.90%). Conversely, the average sales price‐per‐cigarette for premium FMCs in October 2017 (end P3) was +23.06% higher than for mid‐price FMCs, which was greater than the corresponding difference in May 2016 (+20.71%) (start of P1).

## Discussion

Using monthly data from small retailers, we found that the price of leading tobacco products increased during the introduction of standardized packaging in the United Kingdom. Price increases occurred for both RRPs and sales prices for both FMCs and RYO, and for value, mid‐price and premium FMCs. For the 6 months after the legislation became mandatory, there was a small decline in prices. However, based on the new minimum pack sizes, throughout the 18‐month study period the average sales price was estimated to have increased £0.38 for 20 FMCs and £0.91 for 30 g RYO.

Consistent with other UK research, the price‐per‐cigarette and price‐per‐gram increases exceed those expected if only moving in line with tobacco duty escalation 42. For RYO, the March 2017 escalator increased the duty payable per‐gram by approximately £0.012 (from £198.10 per‐kilogram to £209.77) 31. We found that the RRP and sales price‐per‐gram increased above this. For FMCs, the March 2017 escalator increased the duty payable per‐cigarette by approximately £0.012 (from £196.42 to £207.99 per‐1000 FMC) 31. Our results suggest that the RRP and sales price‐per‐cigarette increased above this, particularly for value and premium FMCs. Although the MET was introduced alongside standardized packaging, setting an initial floor price of £6.44 for 20 FMCs (minimum £5.37 of duty plus 20% VAT at sale; equivalent to £0.32‐per‐cigarette) 32, none of the cigarette products monitored had an average sales price‐per‐cigarette below this and were not directly affected. Although increases above duty escalation are not directly attributable to standardized packaging, there is evidence to suggest that the price rises may be an indirect effect of the legislative changes. For example, studies of retail data, interviews with retailers and information in the retail trade press have reported that small retailers used the removal of price‐marking, changes to brand variant name and the new minimum pack sizes as an opportunity to increase sales prices above RRP and increase profit margins 37, 43.

Our findings are consistent with reported increases in RRPs, advertised prices and self‐reported prices paid by consumers following the introduction of standardized packaging in Australia 16, 27, 28, and reported increases in large retailers in the United Kingdom 42. The results do not suggest that the affordability of leading tobacco products increased in response to standardized packaging, contradicting tobacco companies’ claims 8, 9, 10, 11, 13 or that tobacco companies preserved or increased affordability by absorbing duty increases. As we found that price increases were observed for RRPs, which influence the sales prices charged by retailers, this implies that tobacco companies instigated these price rises. This may have been to offset a decline in sales, with research in Australia having found increased attempts to reduce or quit smoking because of standardized packaging 44, 45.

Price increases were observed for value FMCs and RYO products, which offer the most competitive price‐per‐cigarette/gram. Down‐trading is a long‐term trend in the United Kingdom 46 and tobacco companies probably anticipated that this would continue following the introduction of standardized packaging, as happened in Australia 16, 17. The increased prices for value FMCs and RYO products may have been to offset their lower profitability in comparison with mid‐price and premium cigarettes. The small decline in the relative price difference between mid‐price and value FMCs also suggests that tobacco companies tried to incentivise consumers not to down‐trade from mid‐price FMCs in the first place. Premium products, which have a smaller share of the market 47, retained the most expensive price‐per‐cigarette and there was a slight increase in the difference in price between mid‐price and premium FMCs. For products positioned within this segment, in the absence of fully branded packaging to communicate premium characteristics 47, 48, tobacco companies may have been using price as a continued marker of superior quality. It is also possible that tobacco companies over‐shifted costs from duty escalation onto premium products to preserve the affordability of lower‐priced categories 25, particularly as the MET influenced ultra‐low‐priced FMCs (none of which were in our sample).

That projected price declines did not occur as standardized packaging was being phased‐in is a departure from previous tobacco company behaviour in the United Kingdom, where marketing strategies have protected affordability to consumers by under‐ or over‐shifting tax increases 14, 25. The apparent lack of action to preserve the impression of affordability is notable, given that the larger minimum pack sizes required under the legislation created a perceptible increase in overall pack cost to consumers. Nevertheless, our study indicates that price increases were not uniformly applied across FMC price segments, with relative and monetary increases higher for value and premium FMCs compared to mid‐price. Exploring whether this represents deliberate attempts to under‐ or over‐shift duty escalation and the impacts of the MET is an important area for future research. The findings also show a small decrease in price for RYO products and value and mid‐price FMCs once the legislation became mandatory. As the current study only monitored price trends for 6 months after standardized packaging and the MET were mandatory, longer‐term monitoring is needed to understand whether these declines are sustained. Longer‐term monitoring of price trends is also important, as consumers may become increasingly price‐sensitive as the effect of fully branded packaging decays and because the UK Government further increased tobacco duty in November 2017 30, effectively doubling the rate that tobacco duty increased in 2017.

### Limitations and future directions

The results are only representative of price changes in a sample of small retailers, although similar price rises are reported in larger UK retailers 42. Our product sample also only included 20 of the leading tobacco products and their 19 standardized equivalents, and more than half (eight of 15) the sample of cigarettes were ‘mid‐price’. Consequently, our results are not representative of price changes in the wider tobacco market, although they are consistent with UK studies which have monitored a broader product range over a longer retrospective period 42. Further research should explore pricing for smaller products (e.g. 10 FMCs) and specifically analyse the ultra‐value products which will have been affected by the MET 28, 32. The data are only based on monthly average RRPs and sales prices, and data were not collected for sales volume. Such information would help to contextualize how price changes related to reported declines in sales as the legislation was implemented 35, 49. The analyses are also only based on descriptive trends across the sample. Research using more complex statistical techniques to explore how price changes were influenced by area of deprivation for the retailer (e.g. where deprived areas may be more price‐sensitive), by retailer type (e.g. affiliated and non‐affiliated to a symbol group) and whether price changes between periods were significant would be of interest (e.g. interrupted time–series analysis).

As per previous research 27, we analysed changes in price‐per‐cigarette and price‐per‐gram separately. International data suggest that presumed FMC to RYO equivalence varies between country 50 and by method of cigarette production or consumer ability (e.g. hand‐rolled or machine‐produced) 51, 52, with UK research suggesting that consumer‐made RYO cigarettes may contain as little as 0.45–0.55 g of tobacco (i.e. 30 g may produce 40–60 cigarettes) 14, 15. To address this, all analyses of price‐per‐gram for RYO products were presented separately so that the average price‐per‐stick (based on different ratio assumed for RYO stick content) could be understood. Future research, however, could use a combined price‐per‐stick to provide insight into overall affordability. Finally, although the reported price increases exceed those expected through duty escalation, we do not account for the increases in *ad valorem* tax (16.5% on the retail price of FMCs) and VAT (20% on FMC and RYO sales price) that would have been generated by the larger minimum pack sizes (i.e. 20 FMCs cost more than 17, even if offering a more affordable price‐per‐cigarette). Future research calculating changes in prices‐per‐stick and per‐gram after removing all tax components (i.e. gross revenue to retailers and tobacco companies) would be of value, as would research exploring how changes in pack size and the removal of price‐marking influenced price changes.

## Conclusion

The relative cost of leading tobacco products sold by small retailers increased as the United Kingdom phased in standardized packaging. Price increases were observed for both FMCs and RYO and among all FMC price segments, and were larger than increases expected through tobacco duty escalation alone. This contrasts with tobacco companies’ claims that prices would decline in response to the legislation, and the fact that RRPs also increased suggests that tobacco companies were largely responsible for the price rises. This included increases for products that price‐sensitive consumers may have ‘down‐traded’ to (value FMCs and RYO) and continued use of higher prices to distinguish the quality of premium brands. The findings provide important context for research exploring the impacts of standardized packaging and future research exploring tobacco price strategy. Further monitoring is required to understand whether the small declines in price observed once the legislation became mandatory, for both FMCs and RYO, are sustained in the long term.

## Declaration of interests

None.
